# Immunocytes Mediate the Effects of Gut Microbiome on Inflammatory Bowel Disease: Insights From a Mendelian Randomization Study

**DOI:** 10.1155/mi/9956259

**Published:** 2025-08-08

**Authors:** Linbin He, Jianhui Wei, Ziye Li, Suyan Guo, Shanyu Lin, Tingting Wang, Lizhang Chen

**Affiliations:** ^1^Department of Epidemiology and Health Statistics, Xiangya School of Public Health, Central South University, Changsha, Hunan 410078, China; ^2^Hunan Provincial Key Laboratory of Clinical Epidemiology, Xiangya School of Public Health, Central South University, Changsha, Hunan 410078, China

**Keywords:** functional pathways, gut microbiome, immunocyte, inflammatory bowel disease, mediation effect, Mendelian randomization

## Abstract

Observational evidence suggests a complex link between gut microbiota and inflammatory bowel disease (IBD). However, the mechanisms underlying this relationship remain unclear. The present Mendelian randomization (MR) study aims to examine the causal relationships between gut microbiome and IBD (including its subtypes), and to explore potential mediating effects of immunocyte. This MR study utilized the latest genome-wide association study data, which includes 412 gut microbiome features from the Dutch Microbiome Project, a meta-analysis of 731 immunocyte traits, and summary data on IBD from the FinnGen database. The two-sample MR was employed to examine the causal associations, with inverse-variance weighted (IVW) as the main statistical method. In addition, two-step MR was used to explore the mediation effect. Our MR analysis identified the causal effects of 13 microbial taxa, 23 microbial-related functional pathways, and 27 immunocyte traits on IBD. Notably, the dTDP-L-rhamnose biosynthesis pathway is the most significant risk factor for both IBD and its subtypes. After rigorous screening, 10 combinations were examined for mediated effects. This study brings valuable evidence for the relationship between gut microbiome and IBD and the mediating role of immunocyte, providing new insights into the identification of biomarkers and interventional targets for IBD.

## 1. Introduction

Inflammatory bowel disease (IBD) is a group of chronic inflammatory disorders that affect the digestive tract, primarily encompassing ulcerative colitis (UC) and Crohn disease (CD) [1]. The global prevalence of IBD is reported to be around 0.5% to 1%, with an upward trend over the years [2, 3]. IBD not only causes substantial pain for patients but also imposes an elevated economic burden, including medication-related expenses and indirect costs from lost productivity [4]. Current therapeutic strategies usually rely on the widespread use of medical drugs, but often with side effects [5]. Therefore, prevention and early identification of IBD are crucial for alleviating the disease burden. Elucidating the pathogenesis is essential for achieving this goal. However, despite extensive research efforts, the exact causes of IBD remain unclear.

Recent advancements in sequencing technology have highlighted the complicated interplay between the gut microbiota and human diseases, particularly its participation in the development and regulation of diseases [6]. Previous observational studies that sequenced stool samples from IBD patients and controls have found a significant imbalance in the gut microbiome of IBD patients, suggesting that such dysbiosis may be a key factor in IBD pathogenesis [7]. In IBD patients, T cells and associated pro-inflammatory cytokines accumulate in intestinal tissues [8]. Additionally, when the gut microbiome signals innate immunocytes, these cells release inflammatory mediators, activating T and B cells [9]. The Akkermansia can alleviate colitis, possibly by reducing CD8+ cytotoxic T lymphocyte infiltration [10]. This suggests that both the gut microbiome and immunocyte may influence IBD development, and immunocyte may mediate the pathway from the gut microbiome to IBD.

Current studies on IBD are based primarily on the analysis of fecal samples to observe the composition and changes of the gut microbiome [11], or indirect interventions through probiotic supplementation [12], 5-aminosalicylates [13], and other hormonal agents [5]. These studies, while providing important evidence, still have limitations in causal inference. Although randomized controlled trials are ideal for validating causality, such trials are difficult to conduct in humans and are often accompanied by high economic costs due to the diversity of the gut microbiota, the challenges of selecting specific strains, and the complexity of immunocyte regulatory mechanisms. Observational studies are susceptible to confounders that limit the interpretive power of results. Therefore, exploring more appropriate and cost-effective study design to deeply analyze the causal relationship between gut microbiome, immunocyte, and IBD remains an important direction for current research.

Mendelian randomization (MR), an etiological inference method second only to randomized controlled trials in terms of association evidence, uses genetic variation as instrumental variables (IVs) to analyze causality [14, 15]. Since these variants are established at conception, MR is less susceptible to confounders or reverse causation [16]. In recent years, the availability of genome-wide association study (GWAS) data has significantly advanced the application of MR in investigating complex diseases [17], providing unparalleled opportunities to study causal relationships between the gut microbiome, immunocyte, and IBD. Although previous studies have made progress in understanding the links between the gut microbiome and IBD [18, 19], the causal effects mediated by immunocyte remain to be fully explored. In addition, the update of GWAS data emphasizes the necessity of further analysis.

Therefore, this study utilized MR design based on the GWAS summary data to investigate potential causality between the gut microbiome, immunocyte, and IBD. Through these analyses, our goal is to elucidate possible pathogenic mechanisms as well as provide new insights into future prevention and treatment strategies for IBD.

## 2. Methods

### 2.1. Study Design

**Figure 1:**
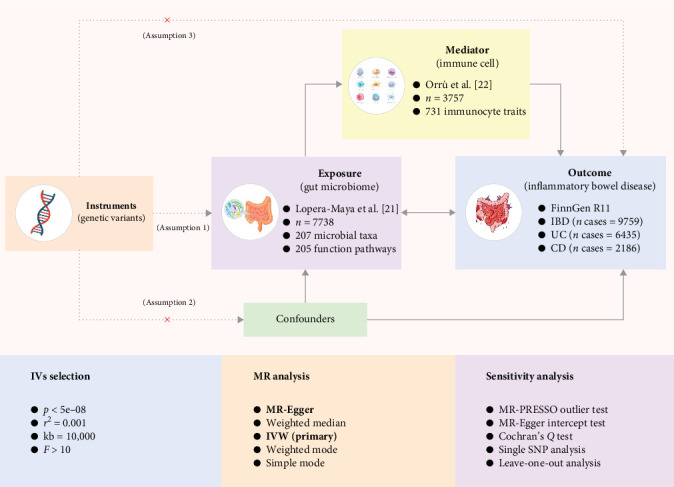
The study design overview. The causal effects and bidirectional causal effects of gut microbiome on IBD. The causal effects of immunocyte on IBD. The mediating analysis of immunocyte in the pathway from the gut microbiome to IBD. CD, crohn disease; IBD, inflammatory bowel disease; IV, instrumental variable; IVW, inverse variance weighted; MR, Mendelian randomization; SNP, single nucleotide polymorphism; and UC, ulcerative colitis.

To ensure methodological rigor, this work was performed in accordance with STROBE-MR lists [20]. The two-sample and two-step MR design was employed to investigate the causal relationships and mediation effect. Corresponding the study design consisted of three parts: analysis of causal relationship between gut microbiome and IBD; analysis of causal relationship between immunocyte and IBD; and mediation analysis of immunocyte in the pathway from gut microbiome to IBD (Figure 1).

### 2.2. Data Source

The data were accessed from the online GWAS database, and participants were of European ancestry. To avoid sample overlap, exposure and outcome data were obtained from separate consortia. Specifically, GWAS summary data for 412 gut microbiome features were acquired from the Dutch Microbiome Project. Lopera-Maya et al. [21] obtained genome-wide association information for 207 microbial taxa by sequencing stool samples from 7738 participants. After screening, the GWAS of 205 pathways representing microbial composition and function was also performed [21]. Immunocyte comprises 731 traits across four features: median fluorescence intensity, absolute cell counts, relative cell counts, and morphological parameters [22]. Information on the heritability and genetic association of immunocyte traits was obtained through a GWAS in a large cohort of 3757 Sardinians [23]. In addition, potential confounders, such as sex, age, and age squared were considered when assessing the associations. Data for IBD, UC, and CD were obtained from the R11 version of the FinnGen database [24]. Detailed information is available in S1.

### 2.3. Screening of Instrumental Variables

We selected IVs by the following criteria [25, 26]: threshold screening (*p* < 5 × 10^−8^); linkage disequilibrium clumping analysis (*r*^2^ = 0.001, kb = 10,000); and exclusion of weak IVs (*F* > 10). The following formulas were applied to calculate the *R*^2^ and *F*-value [27]:

  $$\begin{array}{c} R^{2}=\frac{2\times \beta^{2}\times \text{EAF}\times \left(1-\text{EAF}\right)}{\left(2\times \text{EAF}\times \left(1-\text{EAF}\right)\times \beta^{2}+2\times \text{EAF}\times \left(1-\text{EAF}\right)\times N\times {\text{SE}}^{2}\right)}, \end{array}$$

  $$\begin{array}{c} F=\frac{R^{2}\times \left(N-2\right)}{\left(1-R^{2}\right)}. \end{array}$$

The selection process for immunocyte IVs is identical to that for gut microbiome. To ensure that effect alleles matched between exposure and outcome, the palindromic SNPs were removed during the harmonization step. Then the SNPs related to the outcome were excluded utilizing the LDtrait website (https://ldlink.nci.nih.gov/?tab=ldtrait) [28].

### 2.4. Statistical Analysis

All the data organization and analysis were accomplished in the R environment (version 4.3.3) by employing the “TwoSampleMR” package (version 0.6.0) and the “MendelianRandomization” package (version 0.10.0).

#### 2.4.1. Primary Analysis

The inverse-variance weighted (IVW) approach was adopted as the main statistical method to examine causal associations [29]. A variety of supplementary analysis methods were applied, including MR-Egger, weighted median, simple mode, and weighted mode. The MR findings were considered robust when the directions were consistent across different methods. Other analytical methods, although less powerful than IVW, could support the consistent effect estimate. The odds ratios (ORs) and 95% confidence intervals (CIs) were generated to reflect effect strengths. The association was considered statistically significant when IVW-*p*  < 0.05.

#### 2.4.2. Bidirectional Causality Analysis

After performing the forward MR analysis, bidirectional causality was verified by considering statistically significant gut microbiome features as the “outcome” and IBD as the “exposure.” The *p*-value threshold for IVs selection remained 5 × 10^−8^.

#### 2.4.3. Mediation Analysis

The gut microbiome features and immunocyte traits with significant causal effects on outcome were included in the two-sample MR analysis to clarify whether a significant association existed. Subsequently, the two-step MR analysis was performed to analyze the mediation effect of immunocyte in the pathways from gut microbiome to IBD.

#### 2.4.4. Sensitivity Analysis

The leave-one-out approach and single SNP analysis were used to measure and exclude the influence of individual SNPs on the causal effect. The heterogeneity was assessed utilizing Cochran's *Q* test and illustrated with funnel plots [30]. The MR-Egger intercept test and MR-PRESSO were both employed to detect horizontal pleiotropy [31, 32]. The comprehensive investigation enabled a more in-depth examination of potential sources of bias and provided a more robust assessment of our findings.

## 3. Results

### 3.1. Instrumental Variables Selection

The IVs for 412 gut microbiome features and 731 immunocyte traits were initially obtained through a rigorous selection procedure. The *F*-values of these SNPs are all greater than 10, indicating the absence of weak IVs (S2, S3). In subsequent analyses, these SNPs will be further screened to conform to the criteria for MR analysis.

### 3.2. Causal Effects of Microbial Taxa on IBD

**Figure 2:**
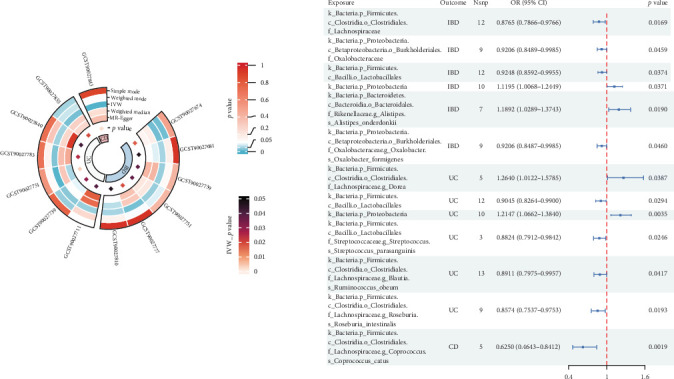
Mendelian randomization results of causal effects between microbial taxa and IBD. CD, Crohn disease; CI, confidence interval; IBD, inflammatory bowel disease; IVW, inverse variance weighted; Nsnp, number of single nucleotide polymorphism; OR, odds ratio; and UC, ulcerative colitis.

The association between microbial taxa and IBD was analyzed based on the IVW approach. Among the 207 microbial taxa, we identified 6, 6, and 1 taxon that were causally associated with IBD, UC, and CD, respectively (Figure 2). In the positive associations, for IBD and UC, the most significant microbial taxa were the species *Alistipes onderdonkii* and the phylum Proteobacteria, respectively. No microbial taxa were identified to be significantly related with CD. Meanwhile, for the negative associations, we found that the family Lachnospiraceae, the species *Roseburia intestinalis*, and the species *Coprococcus catus* showed the most significant associations. Detailed results of all five methods are available in S4–S6. No reverse causal associations were found in the identified combinations.

### 3.3. Causal Effects of Microbe-Related Functional Pathway on IBD

**Figure 3:**
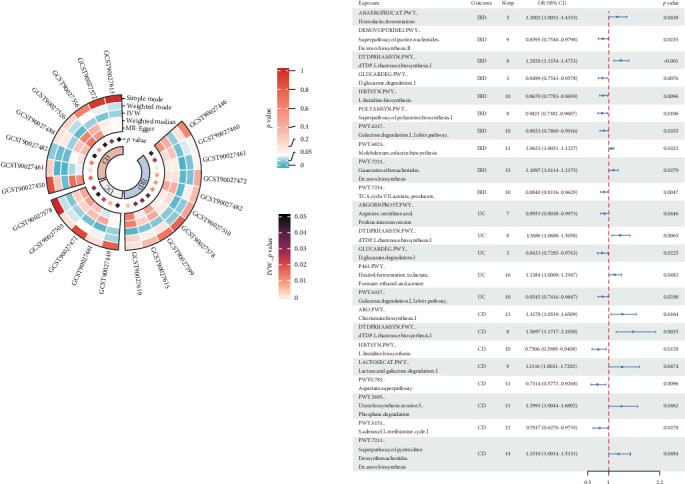
Mendelian randomization results of causal effects between microbe-related functional pathways and IBD. CD, Crohn disease; CI, confidence interval; IBD, inflammatory bowel disease; IVW, inverse variance weighted; Nsnp, number of single nucleotide polymorphism; OR, odds ratio; and UC, ulcerative colitis.

Among the 205 microbe-related functional pathways, we identified 10, 5, and 8 pathways that were causally associated with IBD, UC, and CD, respectively. Notably, the dTDP-*L*-rhamnose biosynthesis pathway showed the most significant positive association in both IBD and its subtypes. For negative associations, the most significant pathways were the TCA cycle VII, the *D*-glucarate degradation I, and the aspartate superpathway, respectively (Figure 3). Detailed results of all five methods are available in S4–S6. Further analysis indicated reverse causality of the two functional pathways in the identified association combinations: superpathway of polyamine biosynthesis I and *D*-galactose degradation I (S13).

### 3.4. Causal Effects of Immunocyte Trait on IBD

**Figure 4:**
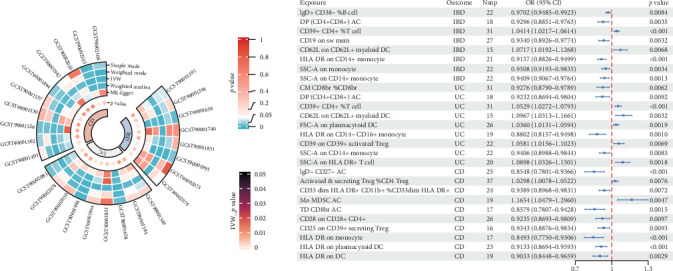
Mendelian randomization results of causal effects between immunocyte and IBD. CD, Crohn disease; CI, confidence interval; IBD, inflammatory bowel disease; IVW, inverse variance weighted; Nsnp, number of single nucleotide polymorphism; OR, odds ratio; and UC, ulcerative colitis.

Based on the IVW approach, we identified 8, 9, and 10 immunocyte traits with significant causal associations with IBD and its subtypes, respectively (Figure 4). For IBD, the most significant positive correlation was with CD39+ CD4+ %T cell, while the negative correlation was with HLA DR on CD14+ monocyte. For UC, CD39+ CD4+ %T cell and HLA DR on CD14− CD16+ monocyte showed the most significant positive and negative correlations, respectively. Finally, for CD, Mo MDSC AC showed the most significant positive association, and HLA DR on monocyte showed the most significant negative association. Detailed results of all five methods are available in S7–S9. These immunocyte traits will be further examined in subsequent analyses to determine if they mediate the effect between gut microbiome and IBD.

### 3.5. Sensitivity Analysis

Various approaches were employed in sensitivity analyses. First, both MR-PRESSO and MR-Egger regression analyses indicated that there were no problems of pleiotropy, including horizontal and directional pleiotropy. Afterwards, heterogeneity was not found in Cochran's *Q* test. The generated funnel plots were roughly symmetric, also indicating the absence of heterogeneity. In leave-one-out analyses, no individual SNPs significantly impacted the effect estimates. The scatter plots visualized the approximate strength and direction of causality. Finally, the effect of each SNP on causality was quantified utilizing single SNP analysis. These analyses confirm the robustness and reliability of our MR analysis in elucidating potential causal relationships (S10–S12).

### 3.6. Mediation Analysis

**Figure 5:**
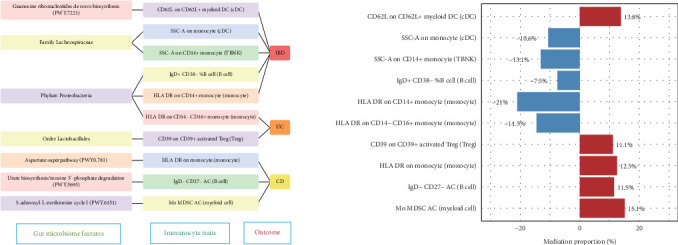
The mediating analysis of immunocyte in the pathway from the gut microbiome to IBD. CD, crohn disease; IBD, inflammatory bowel disease; and UC, ulcerative colitis.

Based on previous analyses, we screened the data: the IVW method had to show significant associations; the causality direction of the other methods had to be consistent; all results needed to pass the sensitivity analysis. We preliminarily identified 10 potential gut microbiome — immunocyte — IBD pathways with mediation proportions ranging from 7.5% to 21% (Figure 5).

In brief, the CD62L on CD62L+ myeloid DC associated with the guanosine ribonucleotides de novo biosynthesis increases the risk of IBD by 13.8%; the HLA DR on CD14+ monocyte associated with phylum Proteobacteria accounts for a 21% reduction in IBD risk. The mediation effect of CD39 on CD39+ activated Treg in the pathway from order Lactobacillales to UC was 0.011, accounting for 11.1% of the total effect; the phylum Proteobacteria mediated by HLA DR on CD14− CD16+ monocyte accounts for a 14.5% reduction in UC risk. The Mo MDSC AC mediated the causal association between *S*-adenosyl-*L*-methionine cycle I and CD, with a mediation proportion of 15.1%.

## 4. Discussion

The latest GWAS summary data was used to provide insight into the causal relationships between 731 immunocyte traits, 412 gut microbiome features, and IBD (including its subtypes). Our research identifies the causal effects of 13 microbial taxa, 23 microbial-related functional pathways, and 27 immunocyte traits on IBD. Although this research is not the first investigation into the association between the gut microbiome and IBD, it has the following novelties: 1) The summary data of the gut microbiome was from a latest large database, which contained 205 microbial-related functional pathways; 2) This work revealed the new microbial taxa and microbe-related functional pathways causally associated with IBD; 3) Importantly, we identified 10 potential immunocyte traits that mediate these effects, improving our comprehension of the complicated mechanisms.

Among the 207 microbial taxa, we identified 6, 6, and 1 taxon that were causally associated with IBD, UC, and CD, respectively. Of these, the species *Alistipes onderdonkii* has been found to be involved in intestinal disorders, such as liver fibrosis and colorectal cancer [33]. However, there is a study suggests that sanghuangporus particularly enriches *Alistipes onderdonkii*, which can produce 5-hydroxyindole-3-acetic acid and ameliorating colitis [34]. Conversely, the family Lachnospiraceae is associated with a lower risk of IBD. Some members of this group have been identified as commercial probiotics [35]. From the metabolite and immunomodulatory point of view, Trichosporonaceae produce short-chain fatty acids (SCFAs), that perform an important part in inducing regulatory T cells [36]. Members of the phylum Proteobacteria are usually present at low levels in healthy individuals, whereas their relative abundance is significantly higher in patients with UC, often leading to imbalances of the gut microbiome [37, 38]. In contrast, the high abundance of the species *Roseburia intestinalis* is related to a lower incidence of UC. Interventions, such as *R. intestinalis* enema, have significantly alleviated colitis symptoms in animal models [39]. In addition, *R. intestinalis* produces butyrate that inhibits colitis by increasing the expression of the *TLR5* gene, and its flagellin induces the immune response and promotes the release of anti-inflammatory factors [40]. Similarly, our research discovered that the species *Coprococcus catus* was linked with a lower risk of CD. Previous studies have shown that the *Coprococcus catus* is highly enriched in healthy controls, with its abundance negatively impacted by pro-inflammatory diets and in CD groups [41, 42].

Among the 205 microbe-related functional pathways, we identified 10, 5, and 8 pathways that were causally associated with IBD, UC, and CD, respectively. The dTDP-L-rhamnose biosynthesis pathway showed the most significant positive association in all disease subtype analysis. This pathway is important for the virulence and viability of numerous harmful bacteria, such as the species Streptococcus, which is higher in the IBD cohort [43, 44]. In addition, we found that TCA cycle VII is associated with a decreased risk of IBD. Lower levels of five TCA circulation-related molecules were found in colonic tissues from patients [45]. As a substrate for the TCA cycle process, acetate ameliorates gastrointestinal conditions by boosting intestinal gluconeogenesis and enhancing the innate immune response through signaling [46]. Furthermore, the aspartate superpathway was linked with the decreased risk of CD. The concentrations of aspartic acid increased in active versus quiescent CD patients [47].

Our study found that the elevation of CD39+ CD4+ %T cell within the immune system correlates with the increased IBD and UC risk. The intestinal immune system maintains immune tolerance to intestinal antigens while responding effectively to invading pathogenic microorganisms by regulating interactions between pro-inflammatory CD4 T cells and tolerogenic regulatory T cells [48]. In IBD patients, activated CD4+ and CD8+ T cells appear in the intestinal mucosa, mediating the inflammatory response [49, 50]. The CD39 is primarily expressed by CD4+ lymphocytes and Treg cell subpopulations, it hydrolyzes pericellular ATP into ADO, which influences Treg immune suppressive activities [51]. In addition, we identified HLA DR on monocyte as a significant factor decreasing the risk of IBD, UC, and CD. The monocytes are normally involved in the inflammatory regulation and immune response [52]. The HLA-DR molecules serve a vital function in antigen presentation and immune regulation [53]. Low expression of HLA-DR in chronic inflammatory monocytes emphasizes its anti-inflammatory role, as noted by Asmussen et al. [54]. In patients with UC and CD, HLA-DR expression was substantially lowered, which is also indicative of the change in the patient's immune status [55, 56].

Through mediation analysis, we identified 10 potential mediating pathways between gut microbiome and IBD. The gut microbiome plays an essential role in the pathogenesis of IBD by regulating the activation of the intrinsic immune system, influencing metabolism of energy, immunological homeostasis, and preserving mucosal integrity. Previous studies have shown that Akkermansia can alleviate colitis, possibly by reducing CD8+ cytotoxic T lymphocyte infiltration [10]. Moreover, gut microbes modulate the metabolism and function of immunocytes through the production of metabolites, including SCFAs, lactate, and bile acid derivatives [57]. The SCFAs modulate protective immunity and tissue inflammation by activating cells via G protein-coupled receptors, leading to the production of chemokine and cytokine. Among them, butyrate promotes Treg development and enhances mucus production by goblet cells, thereby strengthening the mucosal barrier [58–60].

Through these analyses, we gain more information on the gut microbiome features and immunocyte traits associated with IBD. Future approaches targeting gut microbial abundance, metabolite quantities, and modulating functional pathways represent promising strategies for the treatment and prevention of IBD [61]. It has been proposed that targeting specific pathways in immunocytes, such as modulating gut microbial activity to promote the production of metabolites with immunomodulatory functions, could be utilized for the treatment of IBD [62, 63]. We believe that our findings bring valuable evidence for the causal connection between gut microbiome and IBD and the mediating role of immunocyte, providing new insights into the identification of biomarkers and interventional targets for IBD.

However, several limitations must be acknowledged. The data utilized for our analyses were all from European populations, which may restrict the extrapolation of our results. The study utilized summary data, limiting our capacity to analyze causal links in specific subgroups (e.g., sex). Future research could utilize more sophisticated and precise techniques to clarify specific functions and underlying biological mechanisms based on the microbial taxa, functional pathways, and immunocytes our study had identified.

## 5. Conclusion

In this work, we comprehensively examined the causal relationship between the gut microbiome, immunocyte and IBD. Our research identifies the causal effects of 13 microbial taxa, 23 microbial-related functional pathways, and 27 immunocyte traits on IBD. We also identified 10 potential immunocyte-mediated pathways. These newly identified biomarkers may provide the basis for preventive and therapeutic measures in IBD and may also serve as candidate molecules for future mechanistic research.

## Data Availability

The GWAS summary data of 731 immunocyte traits can be accessed at GWAS MRC IEU (ebi-a-GCST0001391-ebi-a-GCST0002121) (https://gwas.mrcieu.ac.uk/). The GWAS summary data of 412 gut microbiome features can be obtained from the GWAS Catalog database (GCST90027446–GCST90027857) (https://www.ebi.ac.uk/gwas/). The GWAS summary data for inflammatory bowel disease (K11_IBD_STRICT), ulcerative colitis (K11_UC_STRICT2), and Crohn disease (K11_CD_STRICT2) can be downloaded from the R11 version of the FinnGen database (https://r11.risteys.finngen.fi/).

## Nomenclature

IBD: Inflammatory bowel disease
UC: Ulcerative colitis
CD: Crohn disease
MR: Mendelian randomization
IV: Instrumental variable
GWAS: Genome-wide association study
SNP: Single nucleotide polymorphism
IVW: Inverse-variance weighted
OR: Odds ratio
CI: Confidence interval
SCFA: Short-chain fatty acid.

## Supporting Information

Supporting Information Table S1. Overview of the source of disease data. Table S2. Details of instrumental variables (IVs)for the 412 gut microbiome features. Table S3. Details of instrumental variables (IVs) for the 731 immunocyte traits. Table S4. The causal effects of gut microbiome on IBD. Table S5. The causal effects of gut microbiome on UC. Table S6. The causal effects of gut microbiome on CD. Table S7. The causal effects of immunocyte on IBD. Table S8. The causal effects of immunocyte on UC. Table S9. The causal effects of immunocyte on CD. Table S10. The sensitivity analysis of gut microbiome and immunocyte on IBD. Table S11. The sensitivity analysis of gut microbiome and immunocyte on UC. Table S12. The sensitivity analysis of gut microbiome and immunocyte on CD. Table S13. Positive results of reverse Mendelian randomization analysis.
